# ABLE: blockwise site frequency spectra for inferring complex population histories and recombination

**DOI:** 10.1186/s13059-018-1517-y

**Published:** 2018-09-25

**Authors:** Champak R. Beeravolu, Michael J. Hickerson, Laurent A. F. Frantz, Konrad Lohse

**Affiliations:** 1 Biology Department, The City College of New York, New York, 10031 NY USA; 2 Department of Evolutionary Biology and Environmental Studies, University of Zurich, Zurich, 8057 Switzerland; 3 The Graduate Center, The City University of New York, New York, 10016 NY USA; 4 Division of Invertebrate Zoology, American Museum of Natural History, New York, 10024 NY USA; 5 Paleogenomics and Bio-Archaeology Research Network, Research Laboratory for Archeology and History of Art, University of Oxford, Oxford, OX1 3QY UK; 6 Institute of Evolutionary Biology, University of Edinburgh, King’s Buildings, Edinburgh, EH9 3FL UK; 7 School of Biological and Chemical Sciences, Queen Mary University of London, London, E1 4NS UK

**Keywords:** Inference, Population history, Composite likelihood, Recombination, Admixture, Orangutan

## Abstract

We introduce ABLE (Approximate Blockwise Likelihood Estimation), a novel simulation-based composite likelihood method that uses the blockwise site frequency spectrum to jointly infer past demography and recombination. ABLE is explicitly designed for a wide variety of data from unphased diploid genomes to genome-wide multi-locus data (for example, RADSeq) and can also accommodate arbitrarily large samples. We use simulations to demonstrate the accuracy of this method to infer complex histories of divergence and gene flow and reanalyze whole genome data from two species of orangutan. ABLE is available for download at https://github.com/champost/ABLE.

**Electronic supplementary material:**

The online version of this article (10.1186/s13059-018-1517-y) contains supplementary material, which is available to authorized users.

## Background

Demographic history has played a major role in shaping genetic variation. However, using this information in an efficient way to infer even very simple models of population history remains challenging: a complete description of the history of genomic samples includes both the ancestral process of coalescence and recombination, as captured by the ancestral recombination graph (ARG). While the ARG is straightforward to simulate, in practice, the number of recombination and coalescent events in any stretch of genome generally exceeds the information (i.e. number of mutations) available to reconstruct them. Thus, it is currently not feasible to perform demographic inference by integrating over all realizations of the ARG that are compatible with a genomic dataset [1].

Current methods dealing with genomic data tackle this problem by making simplifying assumptions about recombination [2]. Methods based on single nucleotide polymorphisms (SNPs) ignore linkage information altogether and make use of the site frequency spectrum (SFS) [3, 4], which is a function only of the expected length of genealogical branches [5, 6]. While computing (or approximating) likelihoods based on the SFS is very fast, much of the information about past demography is sacrificed and recent studies have shown that different demographic histories can give rise to a similar SFS [7].

Other methods seek to use linkage information by approximating recombination, i.e., the sequential transitions between local genealogies along the genome, as a Markov process [8, 9]. Methods based on the Sequential Markov Coalescent (SMC, [10]) are computationally intensive, limited to relatively simple models [11] or small samples [8, 12, 13] and require good genome assemblies which are presently available only for a handful of species.

Multi-locus methods exploit information contained in short-range linkage by assuming that recombination is negligible within short blocks of sequence [14–18]. However, this approach potentially biases demographic inference and still loses information contained in longer range linkage disequilibrium (LD), which is expected to result from historical admixture or drastic changes in population size. While recombination within blocks has been included in multi-locus inference, this currently does not scale up to whole genome data [19]. Interestingly, the few methods capable of jointly inferring recombination (using the SMC) and demography using whole genomes [12, 13] can only analyze a couple of samples or are restricted to specific population histories [20, 21].

To overcome these limitations, we introduce a composite likelihood (CL) framework which is highly flexible both in terms of the demographic histories and data that can be accommodated. We can infer arbitrarily complex demographic histories along with the average recombination rate using multiple whole genomes or genome-wide multi-locus data (e.g., RADSeq) catering to the needs of researchers studying model or non-model organisms, respectively. Our method builds upon an existing analytic approach [16, 18] that partitions the genome into blocks of equal (and arbitrary) size and summarizes the genome-wide pattern of linked polymorphism as a frequency distribution of blockwise site frequency spectra. We refer to this straightforward extension of the SFS as the distribution of blockwise SFS configurations, or simply the bSFS. The bSFS is a richer summary of sequence variation than the SFS, as it retains information on the variation in genealogies contained within the blocks. We use Monte Carlo simulations from the coalescent with recombination to approximate the bSFS. This overcomes the limitations of exact likelihood calculations [18, 22] based on the bSFS by accommodating larger samples of genomes and including recombination within blocks as a free parameter. Our approach is implemented in the software Approximate Blockwise Likelihood Estimation (ABLE) which is freely available (https://github.com/champost/ABLE).

The paper is structured as follows: we first describe how the bSFS can be approximated for samples from single and multiple populations both with and without recombination. The accuracy of our approximation is assessed by comparing it to analytic results for small samples in the absence of intra-block recombination under three different demographic models. We then illustrate the performance of ABLE on real data by analyzing whole genomes from the two species of orangutan (*Pongo pygmaeus* and *P. abelii*) which inhabit the islands of Borneo and Sumatra, respectively [23, 24]. These sister taxa represent an excellent test case as their demographic history has been the subject of several previous analyses [12, 13, 19, 23–25] and the geological knowledge of the Sunda shelf is extensive [26]. The best supported history we infer is a previously unexplored scenario of population divergence (about a million years ago) followed by a discrete pulse of bidirectional admixture which coincides with a cyclical sea-level change in South East Asia [26]. We also obtain plausible estimates for the per-generation genome-wide recombination rate. Finally, we make use of extensive simulations to asses the inferential power of our approach. We explore the ability of ABLE to distinguish between various two-population models and investigate the effects of sample and block size on parameter estimates. We also compare the performance of a small-sample inference with ABLE to that based on the SFS (*∂a∂*i [3]) using larger samples.

## Results

### The blockwise SFS (bSFS)

**Fig. 1:**
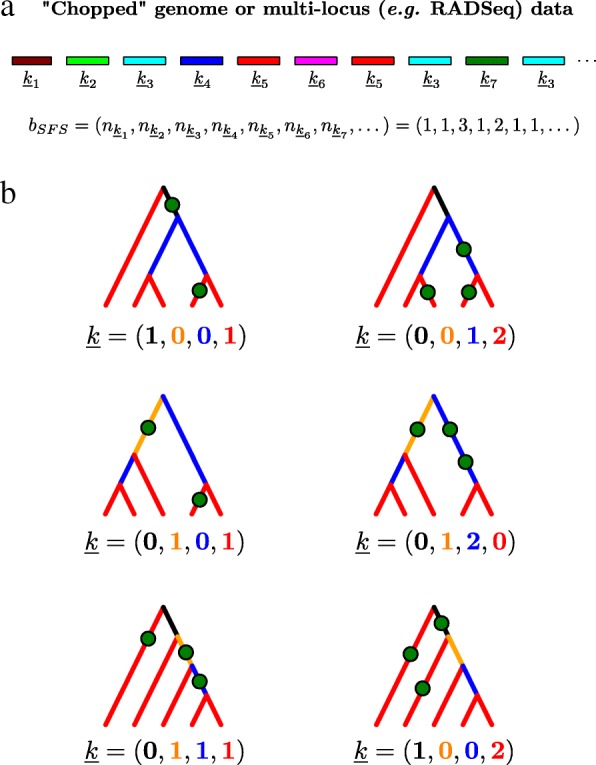
The blockwise SFS (bSFS). **a** The bSFS is computed by partitioning sequences into short blocks, identifying mutation configurations, and noting their respective counts. **b** Example bSFS configurations for a sample from a single population. Genealogical relationships for a sample of size 5 can be generated by three types of topologies (top, middle, and bottom rows). Ignoring information on the phase, the branches can be classified by the number of tips they are ancestral to, i.e., singletons (red), doubletons (blue), tripletons (orange), and quadrupletons (black). Further, mutations on these branches give rise to different bSFS configurations, i.e., vectors $\underline {k}$

Consider a random sample of sequence blocks of fixed length. In practice, such sequence blocks (colored segments in Fig. 1a) may be obtained by partitioning an available reference genome [22, 27] or from reduced representation sequencing strategies, such as restriction site-associated DNA (RADSeq, [28]).

Given a sample of *b* genomes, the polymorphic sites in each sequence block can be summarized by a vector $ \underline {k} $ of length *b*−1 (Fig. 1b). For a single panmictic population, $\underline {k}$ is the SFS of the block and summarizes polymorphic sites within it as counts of singletons, doubletons, etc. Following [22], the bSFS is essentially a frequency spectrum of site frequency spectrum types across blocks (i.e., a histogram of histograms) and can be thought of as a straightforward extension of the SFS that accounts for linkage over a fixed length of sequence block (Fig. 1a).

The bSFS readily extends to samples from multiple populations where the entries of $\underline {k}$ are counts of mutation types defined by the joint SFS [6]. One advantage of the bSFS is that we only require unphased data as mutations are not distinguished based on unique branches but branch classes (singletons, doubletons, etc., see Fig. 1b). In the absence of outgroup information and/or to avoid biases due to errors when polarizing with distant outgroups, the bSFS may be folded. The analytical treatment of Lohse et. al. [18] (see also [22]) assumes non-recombining blocks and uses a recursion for the generation function of genealogies to derive the probability of bSFS configurations for small samples and simple demographic histories involving one or two populations. This allows for a direct comparison with the approximate composite likelihood developed here.

### Approximating the bSFS

The bSFS can be approximated for any given population history while accommodating for intra-block recombination (see the “Methods” section). In summary, we use coalescent simulations to sample the space of blockwise ancestral recombination graphs (ARGs) and compute analytically the probability of observing all bSFS configurations in the data conditional on a particular simulated ARG. Dealing with mutations analytically minimizes both error and computational costs: each simulation replicate contributes to the approximate likelihood of all configurations compatible with it. We used a two-step optimization procedure to hone in on the maximum composite likelihood estimate (MCLE) for a given demographic model (see the “Methods” section).

### Extending the bSFS to arbitrarily large samples

In this paper, we also extend the bSFS by following an obvious and popular [29, 30] strategy that allows analysis of arbitrarily large samples at minimal computational cost by calculating composite likelihoods across subsamples. For instance, depending on the ploidy of the data, a three-population sample containing 24, 50, and 10 genomes, respectively, can now be represented by a cbSFS (or composite bSFS) by subsampling a single genome per population (for haploid data) or every two consecutive genomes for diploid data and so forth. The size of the cbSFS is thus significantly smaller compared to the bSFS of the three-population example. This can be seen as a *projection* of the bSFS (similar to a downprojection of the SFS in *∂a∂*i) from a larger sample size to a smaller sample size. The cbSFS extension in ABLE also improves upon the classic bSFS scheme [16, 27] which was limited to relatively small sample sizes due to the significant increase in size of the latter with sample size (see Table 1 from [18]). Further information on how to generate a cbSFS can be found online (see “Availability of data and materials”).

**Table 1:** Point estimates for the demographic history of orangutan species obtained from 2-kb blockwise data (cf. Fig. 4)

Model	*N*_*A*_	*r*×10^−8^	*T*	*N*_*S*_	*N*_*B*_	*α*_*S*_	*α*_*B*_	4*N*_*A*_*m*_*S*→*B*_	4*N*_*A*_*m*_*S*←*B*_	*T*_2_	*f*_*S*→ *B*_	*f*_*S*← *B*_	*l* *n* *L*
M1	18,200	1.58	387,000										− 907,477
M2	1380	2.06	294,000	22,100	8610								− 891,341
M3	2180	2.09	306,000	21,800	5490	− 0.003	− 0.728						− 891,308
M4	1260	2.11	320,000	22,300	8210			0.025	0.000				− 892,423
M5	1280	1.87	1,807,000	21,600	8850			1.568	2.202	274,000			− 892,225
M6	1420	2.73	816,000	22,400	8910					295,000	0.121	0.267	− 891,139

### Comparison with analytic results

**Fig. 2:**
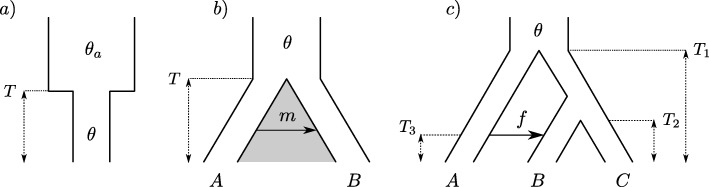
Three demographic models for which ABLE was compared against analytic expectations for the bSFS. **a** A single population with a sudden reduction in *N*_*e*_. **b** IM: isolation between populations A and B followed by continuous unidirectional migration (from A to B) at rate *M* migrants per generation. **c** IUA: isolation between three populations A, B, and C followed by unidirectional admixture of a fraction *f* from A to B. Analytic expectations for these models can be found in [18, 22, 27]

To study how the number of sampled ARGs summarized by the bSFS affects the convergence of the approximate CL to the analytical expectations (i.e. assuming no recombination within blocks), we considered small samples under three simple demographic models: a single population (*b*=4, no outgroup) which doubled in effective size (*N*_*e*_) at time *T*=0.2 (Fig. 2a), a history of isolation between two populations A and B (at time *T*=1.2) followed by continuous unidirectional migration (IM) at a rate *M*=4*N*_*e*_*m*=0.5 migrants per generation from A to B (*b*=2 per population, no outgroup, Fig. 2b), and a history of isolation between three populations (*b*=1 per population with outgroup) with a recent instantaneous and unidirectional admixture (IUA) that transfers a fraction *f* of lineages from population A to B (Fig. 2c). Parameters under the latter model were chosen to correspond roughly to the divergence and admixture history of humans and Neandertals: *f*=0.06, *T*_2_=0.6, *T*_1_=0.15, *T*_*gf*_=0.125 [27]. All times were measured in 2*N*_*e*_ generations. For the sake of simplicity, the models in Fig. 2b, c assume identical *N*_*e*_ for all current and ancestral populations (see also [31, 32]). The analytic solution for the bSFS under these models was previously obtained using an automation for the generating function implemented in *Mathematica* [18, 22, 27].

**Fig. 3:**
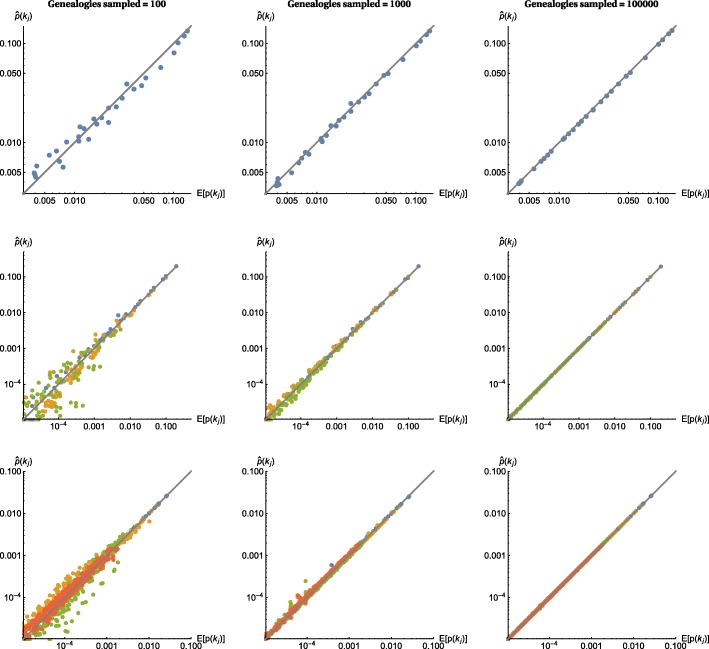
Convergence of the approximated bSFS. The probabilities of bSFS configurations approximated using ABLE converge to the analytic prediction with increasing numbers of simulated genealogies (100, 1,000, and 10,000). Results are shown for models specified in Fig. 2a, b, c (top, middle, and bottom rows) and assuming no recombination within blocks. Block lengths are given in terms of the scaled mutation rate per block and were set to *θ*=0.6, 1 and 2.4 for models shown in Fig. 2a, b, c respectively. For the IM model (Fig. 2b, middle row), bSFS configurations with shared polymorphisms are shown in green, those involving fixed differences in yellow, and those with neither in blue. For the IUA model (Fig. 2c, here in the bottom row), blocks with topology (*A*,(*B*,*C*)), (*C*,(*A*,*B*)), and (*B*,(*A*,*C*)) are shown in yellow, green, and orange, respectively. Topologically uninformative blocks are in blue

The Monte Carlo approximation to the distribution of bSFS configurations matches the analytic prediction extremely well (Fig. 3) even when only small samples of genealogies are used, e.g., 1000 simulated replicates. This is perhaps surprising, given that this sample size is on the same order as the number of unique bSFS configurations. For example, for a sample of *b*=2 from the two populations IM model (Fig. 2b) and counting up to *k*_*max*_=4 mutations per SFS type and block, there are 396 unique bSFS configurations. Interestingly, the probability of bSFS configurations involving fixed differences (Fig. 3; yellow middle row) can be approximated accurately with fewer sampled genealogies than the probability of configurations that include shared polymorphism (Fig. 3; green middle row). This is expected given that we expect greater Monte Carlo error for incongruent genealogies that can induce configurations involving shared polymorphisms because they have lower probability than congruent genealogies (0.16 vs. 0.84 for the IM history we consider). Likewise, for the IUA model, the probability of bSFS configurations involving mutations shared by A and B is harder to approximate than that of (*B*,(*A*,*C*)) configurations (green vs. orange in Fig. 3, bottom row).

### Orangutan analyses

To demonstrate the performance of the ABLE framework on real data, we re-analyzed whole genome data [23, 24] for the two species of orangutan (*Pongo pygmaeus* and *P. abelii*) which inhabit Borneo and Sumatra, respectively (but see [33]). These sister taxa are an excellent test case given that their demographic history has been the subject of several previous analyses [12, 13, 19, 23–25]. We selected a subsample consisting of two diploid genomes per species (i.e., *b*=4 per island) and partitioned the entire autosome into blocks of 2 kb (on average 8.22 SNPs/block). After filtering, a total length of 163 Mb of sequence was retained in the final dataset (see the “Data processing” section for details), which consisted of 36,544 unique bSFS configurations. To investigate the effect of block size on our inference, all analyses were repeated using shorter blocks (500 bp; 9085 unique bSFS configurations) which were obtained by dividing each 2-kb block.

**Fig. 4:**
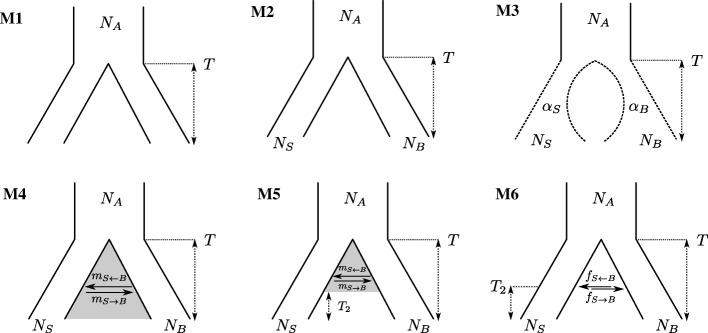
Models of orangutan demography considered in this paper. All models assume a split between two populations at time *T* with an effective ancestral population size *N*_*A*_. In M2–M6, the current population sizes are additional free parameters. M3 allows for exponential growth (or decline) in each population. M4 and M5 assume continuous gene flow since the time of split to the present or to a stopping time *T*_2_, respectively. M6 considers an asymmetric admixture event at time *T*_2_

To facilitate comparison with previous studies (in keeping with the two-species paradigm), we fitted a series of increasingly complex models of divergence with gene flow (Fig. 4) to this data and estimated demographic parameters along with the average genome-wide recombination rate *r* under each model. All demographic models included an instantaneous split at time *T*. We allowed effective population sizes *N*_*e*_ to differ between the two island populations and the ancestral population (M2–M6). Additionally, we considered a model of divergence followed by exponential growth (or decline) in each population given by population-specific growth rates *α* (M3). Asymmetric, bidirectional gene flow was modelled either as a continuous process occurring at a constant rate of *M*=4*N*_*A*_*m* migrants per generation (M4 and M5) or as an instantaneous (bidirectional) admixture pulse affecting a fraction *f* of the admixed population (M6). We considered both an IM model with gene flow from time *T* to the present (M4) and a more complex history of isolation with initial migration (IIM) which assumes that migration ceases at time *T*_2_ (M5) [34]. To convert time estimates (scaled in 4*N*_*A*_ generations) into absolute time, we followed [23] and assumed a generation time of 20 years and a mutation rate *μ*=2×10^−8^ bp ^−1^ per generation.

**Fig. 5:**
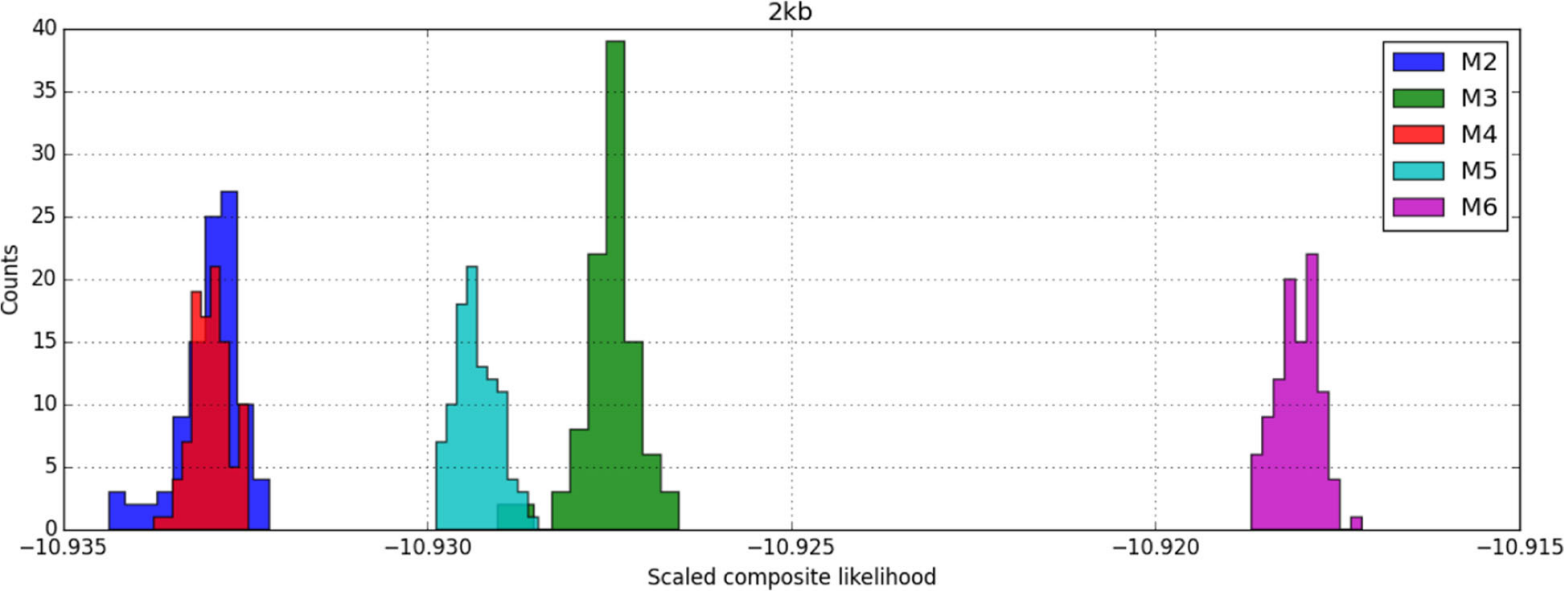
Relative fit of demographic models. Histograms of 100 evaluations of the composite likelihood (CL) using one million ARGs at the MCLE for each model (Table 1). The *x*-axis gives the *per block* CLs, i.e., downscaled with respect to the number of blocks. Models further to the right fit relatively better than those to the left. Model M1 has the worst fit and is not shown (appears much further to the left)

As expected, model support increased with increasing complexity for nested models (i.e., M1 vs. M2 and M4 vs. M5) (Fig. 5 and Table 1). The only exception was the IM model (M4) which did not increase support compared to a strict divergence history (M2). Interestingly, we found greater support for instantaneous admixture (M6) compared to a history of isolation and initial migration (IIM) up to a time *T*_2_ (M5).

Regardless of whether gene flow was modelled as a continuous process (M5) or a discrete admixture event (M6), our analyses reveal greater gene flow from Borneo into Sumatra than in the reverse direction. The maximum composite likelihood estimate (MCLE) under M6 (Table 1), the best supported model, suggests a higher admixture fraction (*f*_*S*←*B*_≈0.27) and no significant admixture in the reverse direction (*f*_*S*→*B*_≈0.12).

Likewise, independent of any particular model, the estimates for the effective size of the Sumatran species were 2.5-fold greater than those for the Bornean species. This is in agreement with previous studies [23] and mirrors the relative diversity in each species as measured by Watterson’s *θ* [35] (*θ*_*W*_=2.19 and 2.91 in 2-kb blocks for the Bornean and Sumatran population, respectively).

To determine the confidence in MCLE under M6, we carried out a full parametric bootstrap by simulating long stretches of sequence under the full ARG and determined 95% confidence interval (CI) as ± 2 SD (standard deviations) across bootstrap replicates (see the “Methods” section for details). The CIs in Table 2 (see also Additional file 1: Figure S1) indicate that we have relatively greater power to infer more recent aspects of orangutan history (*N*_*S*_, *N*_*B*_, and *T*_2_) compared to the time of initial divergence (*T*) and the size of the common ancestral population (*N*_*A*_). While the admixture fraction estimated from Sumatra to Borneo (*f*_*S*→*B*_) was not significantly different from 0, admixture estimates in the reverse direction had much tighter CI which clearly excluded zero.

**Table 2:** Ninety five percent confidence intervals obtained via a parametric bootstrap

Parameter	MCLE ± 2SD
*N*_*A*_	1180–1,670
*r*×10^−8^	2.5– 3
*T*	695,000–936,000
*N*_*S*_	21,200–23,600
*N*_*B*_	8400–9420
*T*_2_	284,000–306,000
*f*_*S*→ *B*_	0–0.21
*f*_*S*← *B*_	0.2–0.33

One hundred datasets were simulated given the point estimates of the 2-kb analysis and model M6 (cf. Table 1). Bootstrap replicates were generated by cutting long (0.5 Mb) contiguous sequences into 2-kb blocks. Confidence intervals were calculated as 2 standard deviations on either side of the maximum composite likelihood estimate

While our study was construed with the long-standing two-*Pongo*-species paradigm, a recent revision of the orangutan history has led to the description of a new species [33]. According to this study, the inferred divergence between *P. abelii* and *P. tapanuliensis* was very ancient (≈ 3.38 Mya), but indirect gene flow is still possible between *P. abelii* and *P. pygmaeus* at more recent time scales (Fig. 3b in [33]), which still warrants the use of our demographic models (Fig. 4). To assess the effect of a third species, we excluded one of the two diploid genomes coming from the *P. tapanuliensis* population (KB9258, see the “Methods” section) and defined a cbSFS sampling scheme consisting of a single diploid per population. The results from this new analysis (Additional file 2: Table S5) confirm the main features of our previous results such as the relatively larger effective population size of the Sumatran population and the relatively lower ancestral population size (Table 1 and Additional file 2: Table S1). However, the cbSFS results from 500-bp blocks halved the divergence time between the two species compared to the normal bSFS results (Additional file 2: Table S1).

### Effect of block length and sample size

We assessed how block and sample size affect ABLE’s ability to infer two-population histories and recombination in two ways. First, we repeated the orangutan analyses using shorter blocks (500 bp). Second, we used simulations to investigate how sampling additional genomes per population affects our inferential power.

#### Block length

Comparing estimates based on 2-kb blocks (Table 1) to shorter 500-bp blocks (Additional file 2: Table S1) suggests that most, but not all, aspects of the inference were fairly robust to block length. As expected, shorter blocks led to a greater uncertainty in model and parameter estimates (Additional file 2: Table S2). Importantly, however, even with 500-bp blocks, M6 was identified as the best fitting model and we found broad overlap in 95% CIs of parameter estimates with the 2-kb analysis.

Both the divergence time *T* and the genome-wide recombination rate *r* were poorly estimated with 500-bp blocks. The 95% CIs of *T* for both 2-kb and 500-bp analyses overlap. In contrast, while the 2kb analyses resulted in fairly stable inferences for *r* (≈2×10^−8^ bp ^−1^ per generation) that agree with recombination estimates for humans [36], the 500-bp estimates were two to four times greater and had very wide 95% CIs (Additional file 2: Table S2).

To test whether our method has any inherent bias to overestimate recombination with shorter blocks, we simulated blockwise data under model M6 using the *r* estimates obtained from the 2-kb data (Table 1). Applying ABLE to these simulated datasets and after taking into account the *Pongo* sampling scheme (i.e., M6 2dp, Additional file 2: Table S3), we noticed no significant overestimation of recombination rates. To test whether gene conversion, a significant feature at such short spatial scales, has an effect on estimates of recombination, we simulated a gene conversion scenario with a crossover to non-crossover rate at 1 and mean conversion tract length at 400 bp (Additional file 2: Table S3). The increase observed in the inferred recombination rate does point to gene conversion as a likely cause underlying the orangutan data and which our inference ignores (see the “Discussion” section).

#### Sample size

As expected, point estimates and power generally improved (Additional file 2: Table S3 and Additional file 1: Figure S2) with increasing sample sizes. While some parameters, in particular *r*, appear non-identifiable with minimal sampling (a single diploid genome per species), all eight parameters of M6 are well estimated with just two or three diploid genomes. We observed a fivefold improvement in accuracy for *r* and up to twofold improvement for demographic parameters when increasing sampling effort from a single to two diploid genomes per population.

Perhaps surprisingly, however, Additional file 1: Figure S2 suggests that for histories similar to that inferred for the two orangutan species, we can expect at best slight improvements in power when adding a third diploid genome per population. Given that analyzing three diploid samples per population almost triples the computation time (Additional file 1: Figure S3), this suggests that (at least in the case of orangutans) analyzing a total of four diploid genomes is a good compromise between information and computational cost.

#### Model misspecification

**Fig. 6:**
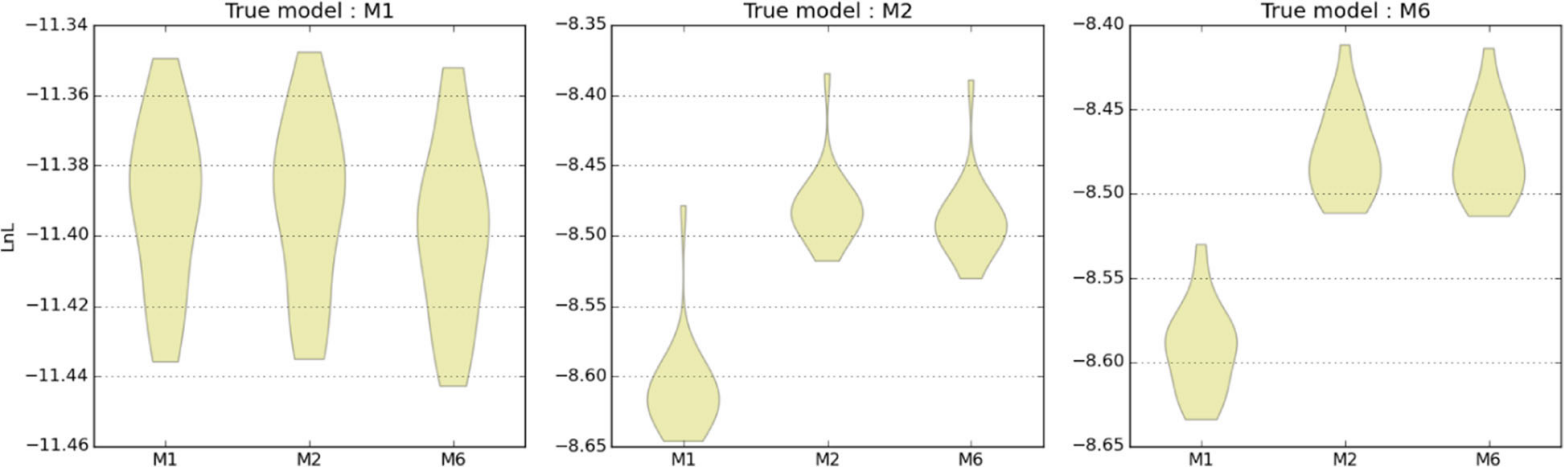
Nested model fit under the true and alternative models. Point LnLs were evaluated at the MCLE under the true and alternative models and for the 20 simulated datasets/model. Higher LnL values (i.e., closer to zero) indicate a better fit to the simulated data

When analyzing real data, the underlying true demography is of course unknown. Thus, an important question is to what extent alternative demographic histories can be distinguished. We evaluated the ability of ABLE to distinguish between three progressively nested models (M1, M2, and M6; see Fig. 4). For each scenarios, we simulated 20 datasets (see Additional file 2: Table S4) and compared the overall fit to the true and alternative models. As expected (given that models were nested), data generated under simple models did not give a better fit to more complex histories (Fig. 6). In contrast, data generated under more complex histories showed a worse fit to simpler scenarios than the truth.

However, given the increased dimensionality of more complex models, the similar LnL values for nested models did not imply that the MCL estimates of demographic parameters under the simpler models were a subset of the corresponding estimates under the more complex models (see Additional file 1: Figure S4, Figure S5, and Figure S6). For instance, given M1 as the true model (Additional file 1: Figure S4), the population split time was largely overestimated under M6 as this model contains a confounding demographic feature, a pulsed admixture event subsequent to divergence. Interestingly, the genome-wide recombination rate was fairly consistently estimated among the various models, while the ancestral population size was consistently underestimated.

To further investigate the ability to correctly identify complex demographies involving post-divergence admixture, we generated 20 simulated datasets under the most complex model considered in the orangutan analysis (M6). We considered nine different divergence/admixture times which varied from 900 to 150 kya and from 600 to 75 kya, respectively, keeping all other parameters fixed (Additional file 2: Table S4) and compared LnL at the true parameter values with MCLE estimated for lower dimensional models M1 and M2. Point LnLs were also calculated for variants of the M1 and M2 models (M1R and M2R, respectively) where the true divergence time was instead replaced by the true admixture time of the simulated dataset (Additional file 1: Figure S7). This analysis illustrates that the ability to identify population divergence and subsequent admixture depends crucially on the interval between these events. When the interval is approximately *T*−*T*_2_<0.3 coalescent units, M2R and M6 become indistinguishable (Additional file 1: Figure S7), which explains the difficulty in distinguishing between M2 and M6 (Fig. 6).

### Comparison between ABLE and *∂a∂*i

Using simulated datasets, we compared the bias and accuracy of ABLE to those of a popular SFS-based method *∂a∂*i [3]. We simulated data under three progressively nested models, M1, M2, and M6 (Fig. 4). We simulated 10 replicate datasets per model (see Additional file 2: Table S4), each consisting of five diploid genomes per population. The *∂a∂*i analyses were based on the SFS from the whole sample, while ABLE used two different sampling schemes. The first was a bSFS for a random subsample of two diploid genomes per population. The second was a cbSFS consisting of all subsamples of two diploid genomes per population.

Despite the fact that ABLE used less than half of the data with the bSFS, it performed as well and in some cases slightly better than *∂a∂*i (Additional file 1: Figure S8, Figure S9, and Figure S10). Overall, *∂a∂*i estimates had less variance than the ABLE estimates, mainly for the ancestral population size, divergence time, and admixture rates. ABLE in general gave less biased estimates of divergence and admixture times than *∂a∂*i and the cbSFS results were always slightly better than the bSFS estimates.

## Discussion

### Orangutan history

The best fitting demographic model (M6) suggests that the two *Pongo* species diverged 650–1000 kya and experienced a burst of admixture around 300 kya. Given the Pleistocene history of periodic sea-level changes in South East Asia [26], such a scenario of secondary contact seems biogeographically more plausible than continuous migration. Reassuringly, our estimates of the divergence time under M6 are consistent with previous estimates based on the SMC [8, 24] and agree well with species splits estimated for other island-endemic mammals in SE Asia [26].

Overall, our results are in general agreement with previous analyses regarding the absence of recent gene flow (< 250 kya) between Bornean and Sumatran orangutans [13]. Likewise, our inference of a larger *N*_*e*_ in Sumatran compared to Bornean orangutans agrees with relative measures of nucleotide diversity and previous analyses using various types of data [12, 19, 23, 25]. While we infer a contraction for the Bornean population under M3, in agreement with the simpler models explored by [25], sampling at finer spatial scales would be required to resolve substructure in both the Sumatran and Bornean populations.

Reassuringly, the time of secondary admixture under M6 agrees with the estimated split time between the two *Pongo* species for simpler models M1–M4 (Table 1) which are similar to those considered by Locke et. al. [23]. Using the joint SFS (*δaδi*, [3]), Locke et. al. [23] estimate a species divergence time of 400 kya, which is somewhat older than our estimate (250–300 kya) under M1–M4. However, a similar difference in estimates has already been noted by the Hidden Markov Model approach of Mailund et. al. [13] (see Supplemental Text S2 in [13]) which models a simplified demography of speciation with continuous gene flow and recombination using whole genome data.

Finally, the recent discovery of a new species (*P. tapanuliensis*, [33]) in Sumatra does not significantly affect our overall results as illustrated by the cbSFS analysis excluding the individual from that population (Additional file 2: Table S5). We do note that the newly inferred effective population sizes are lower than our previous estimates which is to be expected as the removal of the KB9258 individual (from the south of Lake Toba) will have significantly reduced (given its “outlier” status, [37]) the overall polymorphism contained in the cbSFS. In this analysis, which attempts to account for the new species, the genome-wide recombination rate was kept fixed (2×10^−8^/bp/generation) to offset the loss of information. This could explain the lower estimates of the divergence times obtained with the cbSFS from 500-bp blocks.

### Absolute model fit and the effect of selection

Like most demographic inference methods, ABLE assumes selective neutrality. Furthermore, efficient calculation or approximation of the bSFS relies on the assumption that blocks are statistically exchangeable which ignores heterogeneity in mutation and recombination rates.

**Fig. 7:**
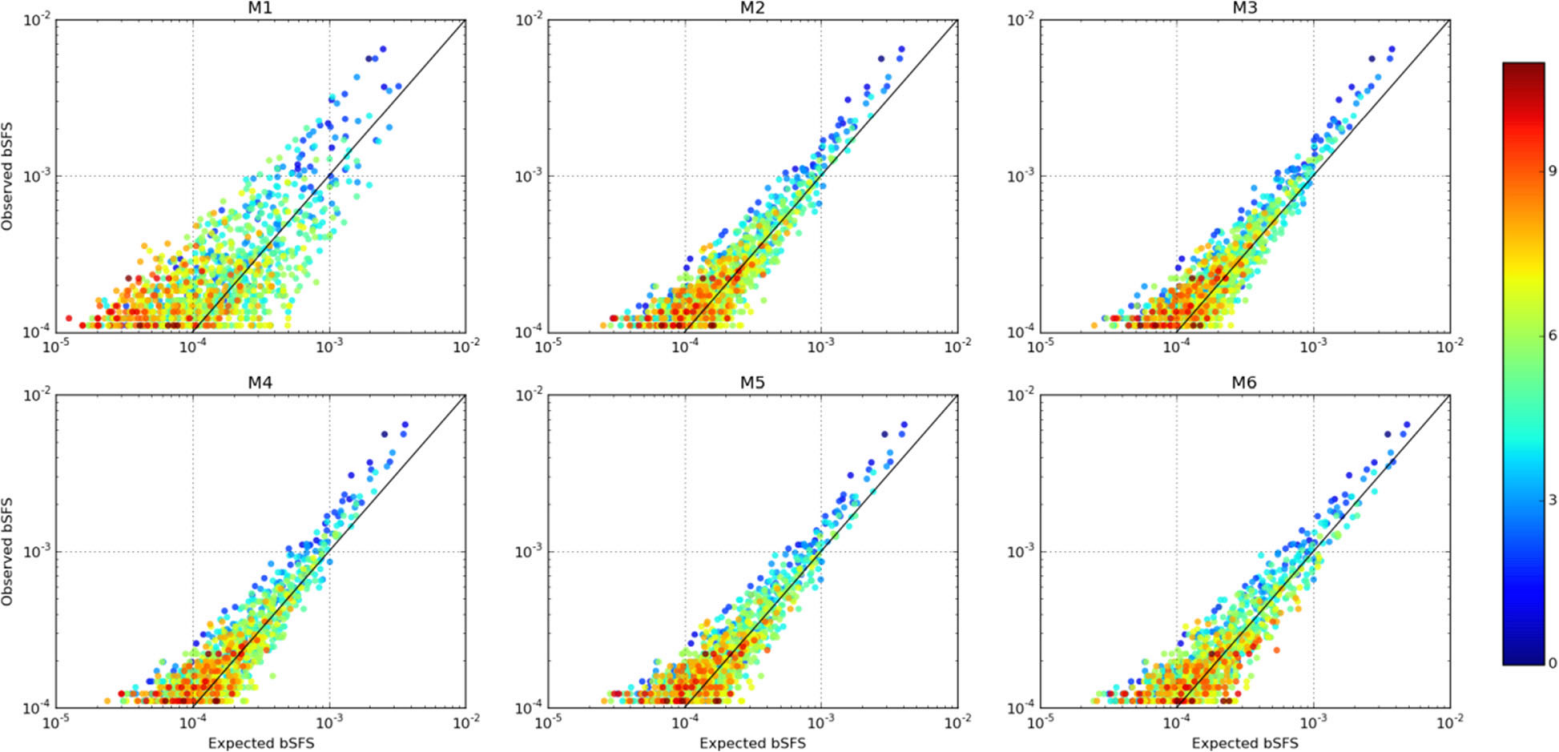
Absolute model fit to the observed 2-kb bSFS for the most common configurations. Each point represents a unique mutational configuration making up the bSFS. The expected bSFS (*x*-axis) was generated with ABLE using 50 million ARGs at the MCLE for each model (Table 1) and plotted against the observed bSFS (*y*-axis) from the orangutan data. The diagonal black line indicates the perfect match between the expected and observed. The colors represent the total number of SNPs contained in each configuration

We can visualize the absolute fit of our demographic model to the data by comparing the observed distribution of bSFS configurations to that expected under M6 (obtained using 50 million simulated blockwise ARGs). If the data were generated entirely by the inferred demographic history, we would expect the most common bSFS configurations to fit this expectation most closely (see Additional file 1: Figure S11). In contrast, Fig. 7 shows that, irrespective of which demographic model we assume, some aspects of the data are poorly captured. In particular, bSFS configurations with few (or no) mutations (shown in blue) are common and overrepresented in the data. This mismatch is compatible with background selection [38] and/or positive selection reducing genetic diversity at a fraction of blocks.

Linked selection may reduce estimates of ancestral *N*_*e*_ under neutral assumptions. Which could explain why we obtained a much smaller effective size for the ancestral population (Table 1 and Additional file 2: Table S1) than previous studies [12, 19, 23, 25], while our *N*_*e*_ estimates for the two current populations agree fairly well [13]. As expected, this signature of linked selection disappears when we consider a bSFS with shorter block size (Additional file 1: Figure S12). It will be interesting to explore the possibility of jointly inferring demography and various forms of selection using the bSFS [39].

### Effect of block length and sample size

An interesting property of the bSFS is that it collapses to the SFS in both the limits of minimal block length (one base) and maximal block length (all data in a single block). At both extremes, all linkage information is lost and so the information contained in the distribution of bSFS types must be maximized at some intermediate block length. While ABLE relies on an arbitrary partitioning of the genome into blocks of a fixed length, recombination breakpoints in the ARG define real “blocks” of sequence that are identical by descent (IBD) with a length distribution that depends on the demographic history in a complex way. Because the distance of IBD blocks is a direct function of the length of genealogical branches, information about different demographic processes is maximized over different physical scales. For example, a burst of recent admixture generates an excess of long blocks that share descent via the admixture event but have different ancestry prior to admixture. The fact that one generally has little prior knowledge about the demography makes it challenging to decide on the most informative block length for a particular dataset.

However, given knowledge of the relative ratio of mutation over recombination events *μ*/*ρ* and assuming that information in the bSFS is maximized if blocks contain on average some small number *x* of IBD tracts, block length can be defined heuristically for a particular *x*. For example, assuming *μ*/*ρ*≈1 for Great Apes, our 2-kb blocks contain on average two to three recombination events within each *Pongo* species (given *θ*_*W*_=2.19 and 2.91 in 2kb blocks for the Bornean and Sumatran populations respectively). A sensible upper (but equally heuristic) bound for the block length is the length at which the number of unique bSFS configurations is maximized which is around 5 kb for the history inferred for the two orangutan species (Additional file 1: Figure S13). However, attempts to partitioning the orangutan data into blocks much longer than 2 kb led to substantial loss of data (given the modest overall coverage), so we did not explore this further.

The fact that ABLE and multi-locus approaches in general rely on a fixed (and necessarily arbitrary) block length is a definite limitation. Thus, an interesting direction for future work would be to integrate CL estimates based on the bSFS over a range of block sizes which should improve the power to infer recent demographic events. A related inference scheme that integrates over a range of window sizes has recently been implemented [20].

Our finding of larger *r* estimates when using shorter blocks for the orangutan data was surprising. Given that our method ignores heterogeneity in both *r* and *μ*, both of which increase auto-correlation across short distances, we expected to find the opposite, i.e., a decrease in *r* estimates for shorter blocks. However, our simulation analysis showed that ABLE gives relatively unbiased estimates of *r* for short (500 bp) blocks when inference was performed with two or more diploid samples per population (Additional file 2: Table S3). A plausible explanation for the large *r* estimates for the orangutan data could be gene conversion because conversion events that span block boundaries are indistinguishable from cross-over events. Results from a simple simulation of a bSFS from 500-bp blocks with gene conversion do highlight this as a probable cause for obtaining higher recombination rate estimates (Additional file 2: Table S3). Furthermore, gene conversion must have a diminishing effect on the bSFS for blocks that are longer than the typical conversion tract length of several hundred bases (see Table 2 in [40]). In the future, it should be possible to use this dependence on block length to develop explicit estimators for gene conversion and cross-over rates.

Even under a complex demography such as M6, our simulation-based power analyses indicate that most demographic parameters can be reasonably recovered with only a single diploid genome per population (Additional file 2: Table S3). Increasing sample size to two diploid genomes more than halved the standard deviation in estimates for some parameters, most notably the recombination rate (Additional file 1: Figure S2). However, a further increase in sample size gave a negligible improvement, despite the considerable computational cost (Additional file 1: Figure S3) involved: the number of unique bSFS configurations increased more than threefold with three rather than two diploid genomes per population. This diminishing return with increasing sample size (in terms of sequences) is a fundamental property of the coalescent [41, 42]: going backwards in time, larger samples in each species are likely to have coalesced down to a small number of lineages (see Fig. 3 in [42]) before the admixture event and so are unlikely to contribute much additional information about older demographic processes.

### The SFS, the bSFS, and the cbSFS

In this paper, we have explored the intuition that using linkage information contained in the bSFS should improve demographic inference compared to the SFS which is only a function of the expected length of genealogical branches [5, 6]. It has previously been shown that the bSFS for a small sample (*n*=5) contains significantly more information about past bottlenecks than the SFS for a large sample (*n*=20, see Fig. 3 in [22]). Likewise, our analysis comparing ABLE with the SFS-based *∂a∂*i [3] for progressively complex subdivided population scenarios (M1, M2, and M6) resulted in improved inferences (with the bSFS) of ancestral population sizes, divergence times, and admixture rates albeit with increased variance in the estimates (Additional file 1: Figure S8, Figure S9, and Figure S10).

However, we only make use of a subset of two diploid genomes for the ABLE analysis compared to the whole sample of five diploid genomes used by *∂a∂*i. This increase in performance can be explained by the fact that the bSFS is a higher dimensional and therefore much richer summary of sequence variation than the SFS [18, 22]. However, this increase in information comes at a computational cost (Additional file 1: Figure S3) and it may be fruitful in general to narrow down parameter space using SFS-based approaches such as *∂a∂*i [3] prior to an ABLE analysis. Finally, the cbSFS scheme provides for an alternative by considering all subsets of the original sample which enables the analysis of arbitrarily large samples at minimal computational cost.

### Limits to inference

While our choice of models was guided by previous knowledge of the demographic history of orangutans [13, 23–25], it remains to be determined what the limits of model complexity and identifiability are with our approach and to what degree the distribution of bSFS patterns overcomes the non-identifiability of the SFS [7, 43, 44]. Unlike analytic likelihood calculations (e.g., [18]), there is no significant increase in computational cost with increasing model complexity when approximating the likelihood for a given point in parameter space. However, searching parameter space carries an obvious and rapidly increasing cost with greater model complexity. Like all approximate likelihood approaches, ABLE requires the user to make careful choices about the number of parameters, the number of genealogies to sample per point in parameter space, and the search bounds for the MCLE, all of which are crucial elements of the optimization strategy [4]. In this regard, we suggest that simple pilot analyses varying some or all of the factors mentioned above (see Additional file 1: Figure S14 and Figure S13) should help to inform the inference strategy.

It is also clear that, independent of the inference approach, the information in the data is finite, so there must be a hard limit on how realistic a history one can hope to infer. Thus, the fact that ABLE can, in principle, be used for fitting any demographic model puts the onus of constraining inference to scenarios that are both statistically identifiable and biologically interpretable on the user. Evaluating the relative fit of simpler nested models is an important sanity check on the limits of information in the data. For instance, our comparison of analyses based on 2-kb and 500-bp blocks (Fig. 5 and Additional file 1: Figure S15, respectively) highlights the limits of our inference scheme for short block lengths.

The inferential approach implemented in ABLE makes use of the coalescent simulator ***ms*** [45] for sampling blockwise genealogies or ARGs. In principle, ABLE can accommodate other simulators and is thus amenable to include additional processes such as linked selection [46, 47]. Another interesting avenue for further research is to apply approximate composite likelihoods based on the bSFS along the genome. Such an approach would not only help improve upon recombination maps for non-model organisms but could also provide a robust framework to identify *outlier regions* of the genome under positive selection and/or affected by introgression from another species.

## Conclusion

We have developed a flexible, efficient, and widely applicable simulation-based approach to simultaneously infer complex demographic histories and average genome-wide recombination rates under the full ARG. This method overcomes the limitations of previous approaches that either ignore recombination [3, 4], use fixed estimates [19], approximate recombination as a Markov process along the genome [8, 11–13], or are limited by the type of population histories they infer [20, 21]. Using the bSFS as a data summary, ABLE captures linkage information at the scale of hundreds to thousands of base pairs and allows researchers to efficiently fit realistic demographic models across the variety of genome scale datasets that are becoming available for a rapidly growing number of species.

The quick asymptotic convergence of the bSFS approximated by ABLE to the expected bSFS under various demographic scenarios (Fig. 3) in the absence of recombination is reassuring and distinguishes our method from related multi-locus approaches that integrate over possible genealogies locus by locus [19]. Furthermore, the extension of the bSFS to the cbSFS now allows the analyses of arbitrarily large samples of whole genomes.

## Methods

### Approximating the bSFS

#### A single population

It is easiest to first consider the simpler case of non-recombining blocks and a sample of *b* genomes from a single panmictic population. We assume an arbitrary population history which is described by a vector of parameters ***Θ***. In the simplest case, ***Θ*** consists of the scaled mutation rate *θ*=4*N*_*e*_*μ*, where *N*_*e*_ is the effective population size and *μ* the mutation rate per site per generation.

The branches of a given genealogy corresponding to our population sample can be partitioned into a vector $ \underline {t} $ whose entries *t*_*i*_∈[ *t*_1_,*t*_2_,…,*t*_*b*−1_] denote the total length of all branches with *i* descendants (Fig. 1b). The probability of observing *k*_*i*_ mutations on a branch class *t*_*i*_ is given by a Poisson distribution with rate parameter *θt*_*i*_>0:

1 $$  p (k_{i} \mid t_{i}) \sim \frac{(\theta t_{i})^{k_{i}} e^{-\theta t_{i}}}{k_{i}!}.  $$

Because mutations occur independently on different branch types, the joint probability of seeing a specific configuration $ \underline {k}_{j} = \{k_{1,j},k_{2,j},\ldots,k_{b-1,j}\} $ in a sequence block *j* and for a given branch length vector $ \underline {t} $ is then a product of Poisson distributions

2 $$  p(\underline{k}_{j} \mid \underline{t}) = \prod_{i=1}^{b-1} p(k_{i,j}\mid t_{i}).  $$

The likelihood $\mathcal {L}(\boldsymbol {\Theta })$ at a point in parameter space $ \boldsymbol {\Theta } \in \mathbb {R}^{+}$ is calculated as

3 $$  \mathcal{L}(\boldsymbol{\Theta}) \propto p(\mathcal{D}\mid\boldsymbol{\Theta}) = \sum_{\mathcal{G}}p(\mathcal{D}\mid\mathcal{G},\boldsymbol{\Theta})p(\mathcal{G}\mid\boldsymbol{\Theta}),  $$

where $ \mathcal {G} $ is the (unknown) genealogy and $ \mathcal {D} $ the data [48]. Summarizing genealogies $ \mathcal {G} $ by $ \underline {t} $ and $ \mathcal {D}$ by $ \underline {k}_{j} $ and drawing $ \mathcal {M} $ random samples of $ \underline {t} $ from $ p(\underline {t}\mid \boldsymbol {\Theta }) $, the Monte Carlo approximation of Eq. 3 can be obtained

4 $$  \hat{p} (\underline{k}_{j} \mid \boldsymbol{\Theta}) \approx \frac{1}{\mathcal{M}}\sum_{d=1}^{\mathcal{M}} p(\underline{k}_{j} \mid \underline{t}_{\,d},\boldsymbol{\Theta}).  $$

In theory, each block in a dataset might have a unique bSFS configuration. In practice, however, for short blocks spanning a handful of SNPs (e.g., < 10), the number of observed bSFS configurations will be much smaller than the number of blocks. Assuming that blocks are equivalent and independent, that is, they have the same length, per base mutation and recombination rates and are unlinked, we can summarize the entire genome into *blockwise data* (Fig. 1a) by counting the number of each unique bSFS type $ n_{\underline {k}_{j}} $. Thus, the approximate joint composite log likelihood for a sample of *n* genomes is given as

5 $$  \ln (\mathcal{L}(\boldsymbol{\Theta})) =\sum\limits_{\underline{k}_{j}} \ln (\hat{p}(\underline{k}_{j})) n_{\underline{k}_{j}}.  $$

#### Multiple populations

The Monte Carlo approximation detailed above extends to the joint bSFS [6, 18] for multiple populations. Assuming a sample from *X* populations, the (unfolded) joint bSFS defines $ \left (\prod _{x=1}^{X} b_{x} + 1\right) - 2 $ site types, where *b*_*x*_ denotes the number of genomes sampled from population *x*. Some branches will be specific to a single population, while others are shared between populations. Thus, the vectors $ \underline {t} $ and $ \underline {k} $ have entries corresponding to the joint bSFS. Note that one specific configuration which we denote as *k*_0_ refers to monomorphic blocks.

#### The ancestral recombination graph

In the presence of recombination, the ancestry of a sequence block is described by the *ancestral recombination graph*$ \mathcal {A} $ [1] which can be partitioned into a set of marginal genealogies corresponding to the non-recombining segments that make up the block [49]. Here, ***Θ*** consists of the scaled mutation rate *θ* and the scaled recombination rate *ρ*=4*N*_*e*_*r*, where *r* is the recombination rate per site per generation. For a given $ \mathcal {A} $, let *S* be the number of non-recombining blocks with respective (sequence) lengths *w*_1_,*w*_2_,…,*w*_*S*_ such that the size of the sequence block $ L = {\sum \nolimits }_{p=1}^{S} w_{p} $. Let $ \underline {t}_{\,p} $ be the marginal branch length vector for each non-recombining segment *p*. The total length of the *i*th branch class over the graph $ \mathcal {A} $ is then given by

6 $$  t_{\{i,\mathcal{A}\}} = \frac{1}{L} \sum\limits_{p=1}^{S} w_{p} t_{\{i,p\}}  $$

Following Eq. 1, we can write the joint probability of observing a specific bSFS configuration over the entire recombining block as $ p(k_{\{i,\mathcal {A}\}} \mid t_{\{i,\mathcal {A}\}}) \sim Poisson(k_{\{i,\mathcal {A}\}};\theta t_{\{i,\mathcal {A}\}}) $ (analogous to Eq. 2). Drawing $ \mathcal {M} $ random samples of $ \mathcal {A} $ from $ p(\mathcal {A}\mid \boldsymbol {\Theta }) $ and replacing $ p(\underline {k}_{j} \mid \underline {t}_{\,d},\boldsymbol {\Theta }) $ with $ p(\underline {k}_{\,\mathcal {A}_{j}} \mid \underline {t}_{\,\mathcal {A}_{j}},\boldsymbol {\Theta }, \rho)$ in Eqs. 4 and 5 give the approximate likelihood for a point in parameter space $\boldsymbol {\Theta },\rho \in \mathbb {R}^{2+}$ (see also [19]). However, note that $ \boldsymbol {\Theta } \in \mathbb {R}^{2+} $ can be too restrictive a criterion for some parameters of complex demographies such as coefficients of exponential population expansion/contraction where $ \alpha _{S},\alpha _{B} \in \mathbb {R} $ (see Fig. 4).

### Implementation

The ABLE implementation includes a seamless integration (invisible to the user) of the simulator ***ms*** [45] for sampling genealogies from $ p(\mathcal {G} \mid \boldsymbol {\Theta }) $ or $ p(\mathcal {A} \mid \boldsymbol {\Theta }) $. Crucially, for each simulated genealogy, we only record the total branch lengths of all SFS classes $ t_{\{i,\mathcal {A}\}} $ in each ARG. This is a sum over marginal genealogies contributing to the ARG, each weighted by its length. From these, we can tabulate the probabilities (conditional on $ \mathcal {G} $) of all bSFS patterns compatible with that ARG. This task is extremely efficient compared to previous *multi-locus* methods that sample $ \mathcal {G} $ separately for each locus (see [19, 50]).

Note that ABLE differs from previous, analytic calculations based on the distribution of the bSFS configurations in an important way. Lohse et al. [18] tabulate probabilities of all bSFS configurations up to a maximum number of mutations (*k*_*max*_) in each category and lump all configurations >*k*_*max*_ mutations.

7 $$  p (k_{i} > k_{max} \mid t_{i}) = 1 - \sum\limits_{k_{i}=0}^{k_{max}} p (k_{i} \mid t_{i}),  $$

and

8 $$  \begin{array}{lr} p (k_{i} = 0 \mid t_{i}) = 1\\ p (k_{i} > 0 \mid t_{i}) = 0 \end{array}, \forall\,t_{i} = 0.  $$

Bounding the table of mutational configuration in this way makes analytic computations feasible and ensures that the table of probabilities sums to unity. However, choosing *k*_*max*_ involves a trade-off between computational efficiency (low *k*_*max*_) and information (high *k*_*max*_). In contrast, ABLE only computes probabilities for mutational configurations that are observed in the data without setting any bounds on the space of possible configurations.

ABLE is implemented in C/C++, follows closely the command-line structure of ***ms*** [45] along with a brief configuration file with additional instructions, and is freely available for download from https://github.com/champost/ABLE.

### Data processing

Raw reads were downloaded from the NCBI Sequence Read Archive (SRA) for two individual genomes each from Borneo (B) and Sumatra (S): KB5405 (B, male, SRS009466), KB4204 (B, male, SRS009464), KB9258 (S, female, SRS009469), and KB4361 (S, female, SRS009471). Mean depth of coverage was between 7.25 and 8.06 per individual. The alignment was performed using BWA-MEM [51] v0.7.5, with a re-alignment step using GATK v.3.3 [52]. For each sample, we estimated a 95% depth of coverage interval using BEDTools [53]. To call genotypes, we used a simple approach [54, 55]: we generated pileup files using samtools v1.3 “mpileup” (0.1.19) [56] with default settings. Pileup files were then filtered, for each sample, using the following criteria:

- Minimum depth of coverage ≥ 4 reads with mapping quality ≥ 30
- Excluded all sites in region of high DoC (top 5%) (coded as N to avoid copy number variant)
- Excluded all sites within 5 bp of an indel (coded as N to avoid indel misalignments)
- Only bases with quality ≥ 30 within reads with mapping quality ≥ 30 were used.
- Minimum fraction of reads supporting heterozygous (variant allele frequency [VAF] ≥ 0.2). Sites that did not pass this criterion (0< VAF <0.2) were coded as missing (N).

Thereafter, we binned the genome into non-overlapping blocks of fixed length *l*=2 kb and sampled the first 0.8×*l*=1600 bases in each block that passed filtering in all individuals (a python script is available online, see “Availability of data and materials”). Blocks with fewer bases post filtering were excluded. The 500-bp dataset was generated by partitioning each post-filtered 1.6-kb block into four blocks of equal size. The 500-bp and 2-kb block datasets used in this study are available for download from the aforementioned website.

### Optimization

Because ABLE approximates the likelihood function (Eq. 5) using Monte Carlo simulations—which induces some variability in the CL obtained (Additional file 1: Figure S14)—algorithms based on the gradient of the CL surface (e.g., [3, 9]) are not reliable [4]. In addition, due to the possibility of multiple local optima in the likelihood surface, we adopted a two-step search heuristic.

We initially searched parameter space between broad, user-specified non-linear bounds as part of a *global search* step. Search bounds during this step spanned several orders of magnitude for all parameters. Upper bounds of some parameters were set on the basis of simple data summaries, e.g., effective population sizes were bounded by Watterson’s *θ*_*W*_ [35]. Fifty thousand ARGs were used to approximate the CL at each point in 10 replicate global searches. These were then used to set narrower bounds for a *local search* based on 500,000 ARGs/point which was repeated 20 times. In Table 1 and Additional file 2: Table S1, we report the best MCLEs whose likelihoods have been evaluated using 1M ARGs. For some models for which replicate local searches did not converge sufficiently, a second round of local searches was used.

ABLE employs several search algorithms implemented in the Non-Linear optimization library (NLopt version 2.4.2, [57]). Both global and local searches used the improved penalization provided by the Augmented Lagrangian algorithm [58] to navigate the non-linear delimitation of parameter space. A controlled random search with rules for the evolution of a population of points given by the Local Mutation algorithm [59] was used for global searches. Local searches used the Subplex algorithm [60], a variant of the Nelder-Mead simplex with start points that were randomly chosen within the parameter bounds set by the global searches.

Finally, tolerances for terminating MCLE searches were determined by probing the CL surface (e.g., Additional file 1: Figure S14). The command lines and configurations used to analyze the orangutan data are available online (see “Availability of data and materials”).

### Parametric bootstrap and simulation analysis

While the CL is a statistically consistent estimator of demographic parameters and recombination (in the limit of large data, [61]), it suffers from severe overconfidence because correlations between blocks due to their physical linkage are ignored. To obtain meaningful measures of confidence, we conducted a full parametric bootstrap under the best fitting model (M6) and parameter estimates (Table 1). We simulated 100 replicate datasets of 164 Mb each using a modified version of ***ms*** [45] (using SimLinkedBSFS; see “Availability of data and materials”) and under the best model (i.e., M6) and MCLE (Table 1 and Additional file 2: Table S1). Blocks in each dataset were assumed to be completely linked (given our estimate of per site *r*) across 0.5-Mb stretches of sequence. These simulations represent an extreme case of linkage and are thus conservative. Indeed, our real data contain large gaps between blocks especially due to the highly repetitive nature of the orangutan genome. As we wish to know the *local variability* of the bootstrap inferences around the MCLE obtained from the orangutan data, we only carried out local searches for each bootstrap replicate (using the boundaries and step sizes obtained in the analysis of real data, see above).

The simulation-based power test exploring the effect of sample size (one to three diploid genomes per population) was based on inferences using simulated data followed by a full parametric bootstrap. Given the computational effort required (see Additional file 1: Figure S3), we restricted our study to 500-bp blocks with values for the demographic parameters chosen to represent the results inferred from the real data under M6 (Table 1 and Additional file 2: Table S1). Parametric bootstrap datasets were generated with linkage (under the full ARG) exactly analogous to the bootstrap in the real data analysis. An additional dataset (using 500-bp blocks) was simulated with gene conversion to check whether an inference with ABLE (which ignores non-crossover events) results in higher recombination rate estimates. This dataset was generated with the crossover to non-crossover rate at 1, mean conversion tract length at 400 bp, and keeping all other demographic parameters the same as above (Additional file 2: Table S3).

### Evaluating nested model misspecification

To evaluate model misspecification, we compared the overall fit of several models to a dataset simulated under a specific model. Thus, datasets were generated under three two-population demographic scenarios M1, M2, and M6 (4). For each scenario, two diploid genomes per population sample were simulated (using ***ms*** [45]); each of which had a size of 200 Mb (made up of independent 1-Mb blocks) and two diploid samples/population. The values used for the simulation can be found in Additional file 2: Table S4. Under any given model, the MCLE search strategy consisted of three global searches of the parameters with successive refinement of the parameter bounds and finally a local search. The final likelihoods were evaluated using 1M genealogies.

Assuming that the parameter values used to simulate data would have been close to the inferred global maximum under both the true and alternative models, we also attempted an illustration of model choice with ABLE by comparing LnLs under M1, M2, and M6 in the tricky situation when M6 is the true model (Additional file 1: Figure S7). Twenty datasets were simulated under model M6 and nine different split/admixture times. Split times varied from 900 to 150 kya whereas admixture times varied from 600 to 75 kya and sample sizes were the same as in the previous section. Model fit was assessed using point LnLs calculated at the true parameter values of each simulated dataset which meant using only a subset of those values for the lower dimensional models M1 and M2. Point LnLs were also calculated for variants of the M1 and M2 models (M1R and M2R, respectively) where the true split time was instead replaced by the true admixture time of the simulated dataset.

### Comparison between *∂a∂*i and ABLE

We compared (under models M1, M2, and M6) the performance in terms of parameter inference between *∂a∂*i [3] and ABLE when the latter uses either a single subset of every simulated dataset or every subset of all simulated datasets. Akin to the previous section, we simulated 2 population demographic scenarios under M1, M2, and M6. Each simulation consisted of five diploid genomes per population sample and each genome was made up of 1M 2-Kb blocks (i.e., 200 Mb in total size). A total of 10 datasets were simulated for each of the three scenarios (see Additional file 2: Table S4). Each dataset was either summarized as the folded SFS (for a subsequent *∂a∂*i analysis), the folded bSFS by randomly sampling two diploid genomes from each population, and the folded cbSFS by sampling *all* diploid genomes from each population (the latter two for a subsequent ABLE analysis).

Parameter inference under M1 and M2 for both *∂a∂*i and ABLE analyses was performed using 10 independent (local) searches on each simulated dataset. For the M6 scenario, ABLE analyses followed a global search with successive refinement due to the high dimensional search space while *∂a∂*i analyses were consistent with its previous strategy. Python scripts defining the models M1, M2, and M6 to facilitate a *∂a∂*i analysis and the bioinformatic pipeline for obtaining a bSFS/cbSFS have been made available online (see “Availability of data and materials”).

## Additional files

Additional file 1 **Figures S1–S15.** Supplementary figures. (PDF 1193 kb)

Additional file 2 **Tables S1–S5.** Supplementary tables. (PDF 120 kb)
