# Supplementary material for: ABLE: blockwise site frequency spectra for inferring complex population histories and recombination
Source: Genome Biol. 2018 Sep 25;19:145. doi: 10.1186/s13059-018-1517-y (PMC6156964; doi:10.1186/s13059-018-1517-y)

ABLE: blockwise site frequency spectra for inferring complex population histories and recombination

Supplementary figures S1 - S15

Fig. S1 Correlations between the parametric bootstrap estimates obtained for the 2kb dataset (Table 2). The scatter plots in the lower and upper half of the figure (are symmetrically identical) show the pairwise correlations among parameter estimates under M6. Density plots for each parameter are respectively given along the diagonal.

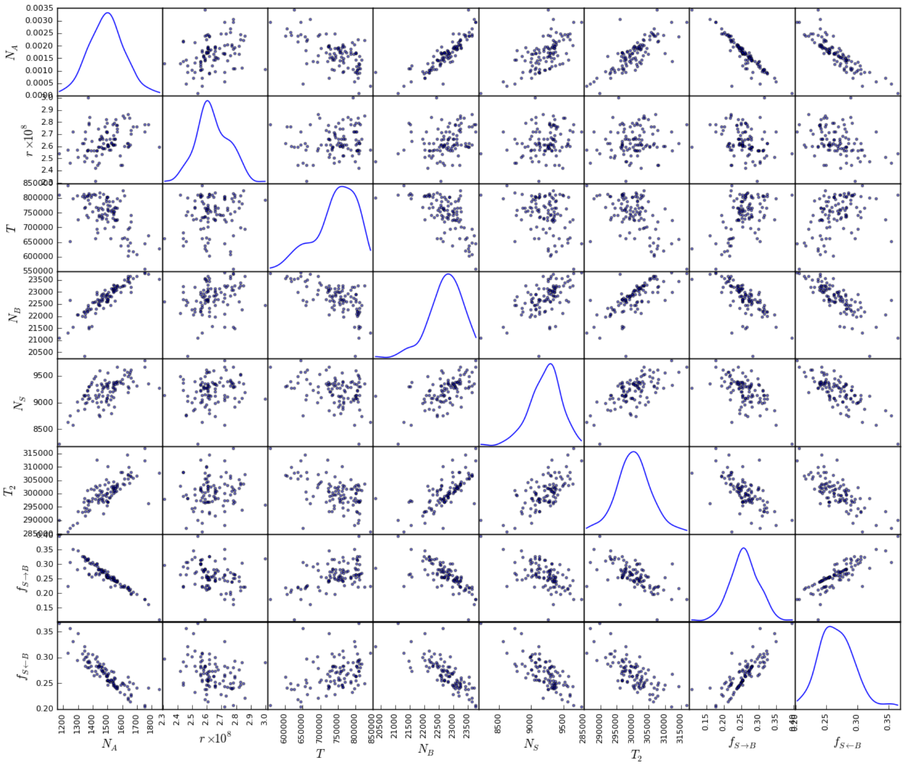

Fig. S2 Parametric bootstrap results for model M6 and varying sample size. The x-axis indicates the number of diploid genomes per population. Accuracy for each parameter is given as the base-2 logarithm of the relative error (*i.e.* estimated value divided by the truth): 0 (red line) corresponds to the truth, increments of +1/-1 to two-fold over- or under-estimates on the coalescent time scale. The black dots show the inferred point estimates for the 1, 2 or 3 diploid genomes per population schemes given in Table S3. The blue dots correspond to the inferred point estimates on data simulated with gene conversion (Table S3).

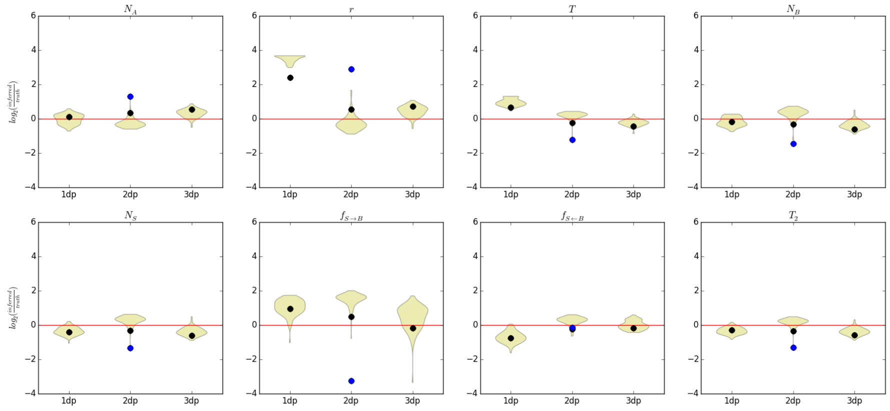

Fig. S3 Computation times with varying sample and block sizes. Simulated datasets were generated by varying the number of diploid genomes per population under model M6 (for values used, see Table S3). Two types of block sizes were considered (500bp and 2kb) by keeping the whole genome size fixed at 200Mb. A single likelihood was evaluated at the true MCLE for each sampling configuration and block size by varying the number of sampled ARGs (x-axis). Times have been approximated to the nearest second.

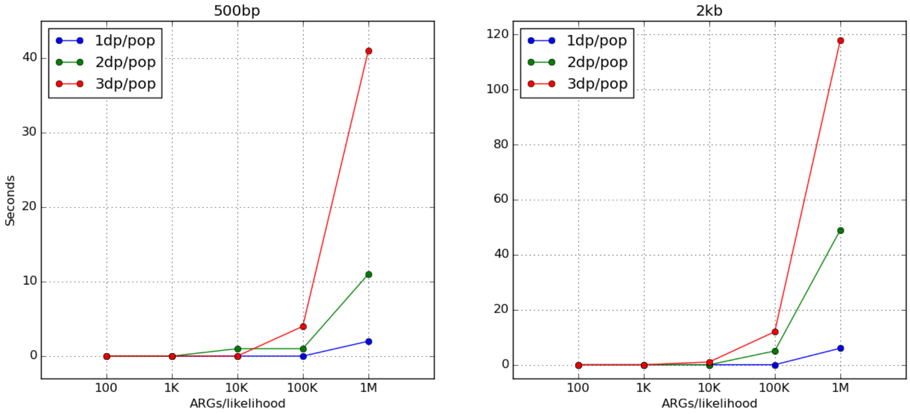

Fig. S4 Parameter inference under the true and alternative models. The y-axis represents inference accuracy as given as the base-2 logarithm of the relative error (*i.e.* estimated value divided by the truth): 0 (red line) corresponds to the truth, increments of +1/-1 to two-fold over/under-estimates respectively.

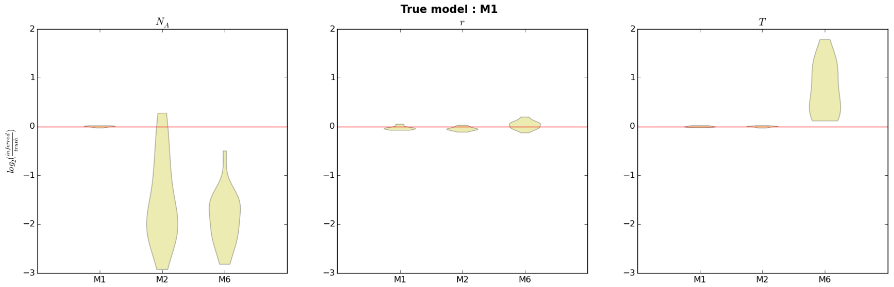

Fig. S5 Parameter inference under the true and alternative models. The y-axis represents inference accuracy as given as the base-2 logarithm of the relative error (*i.e.* estimated value divided by the truth): 0 (red line) corresponds to the truth, increments of +1/-1 to two-fold over/under-estimates respectively.

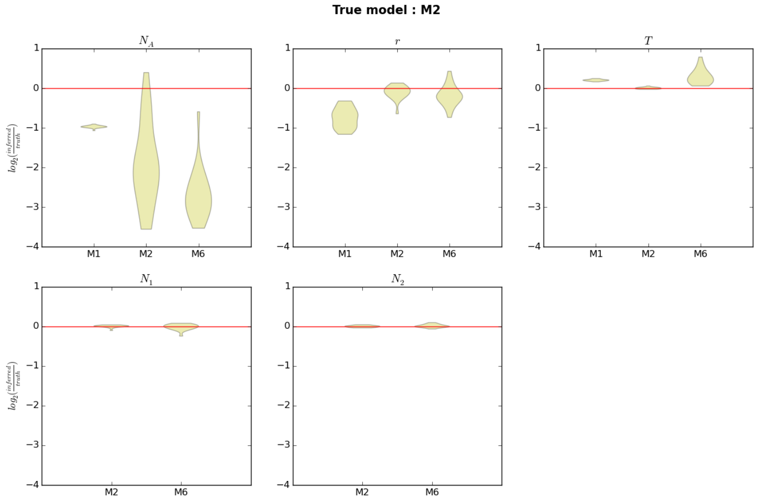

Fig. S6 Parameter inference under the true and alternative models. The y-axis represents inference accuracy as given as the base-2 logarithm of the relative error (*i.e.* estimated value divided by the truth): 0 (red line) corresponds to the truth, increments of +1/-1 to two-fold over/under-estimates respectively.

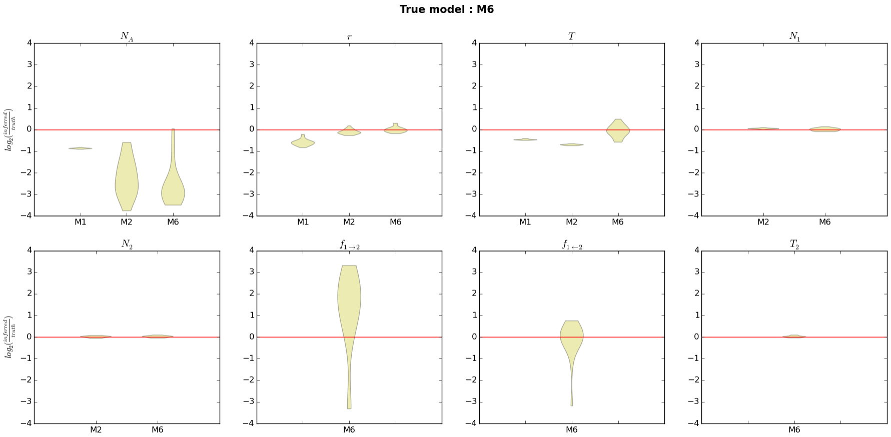

Fig. S7 Nested model fit under the true (M6) and alternative models. Point LnLs were evaluated at the obtained MCLE under the true (M6) and alternative models and for the 20 simulated datasets/model. M1R & M2R represent alternative (to M1 & M2 respectively) scenarios where the divergence time between populations (T) was set to be that of the true admixture time(*i.e.* T_2). Above each plot, the true split and admixture times are given in a $T : T_2$ format and given in parenthesis is $T - T_2$ in coalescent units. Higher LnL values (*i.e.* closer to zero) indicate a better fit to the simulated data.

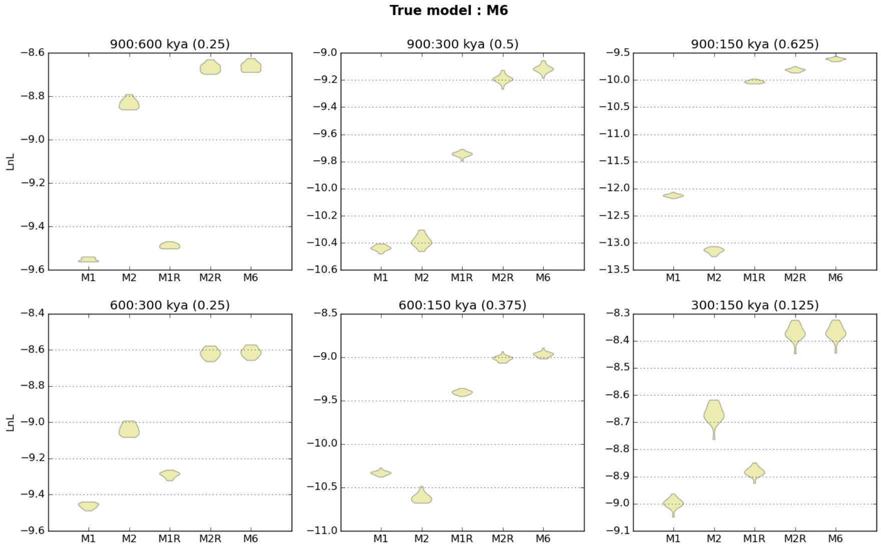

Fig. S8 Comparison of ∂a∂i and ABLE under model M1. The y-axis represents inference accuracy as given as the base-2 logarithm of the relative error (*i.e.* estimated value divided by the truth): 0 (red line) corresponds to the truth, increments of +1/-1 to two-fold over/under-estimates respectively.

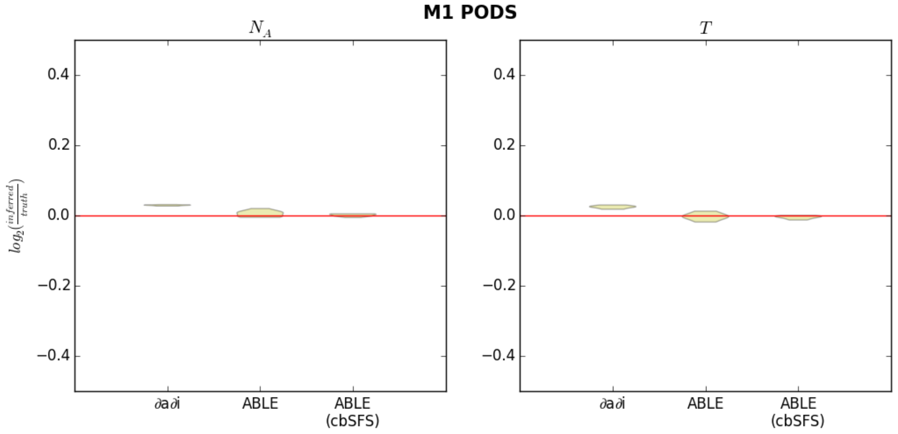

Fig. S9 Comparison of ∂a∂i and ABLE under model M2. The y-axis represents inference accuracy as given as the base-2 logarithm of the relative error (*i.e.* estimated value divided by the truth): 0 (red line) corresponds to the truth, increments of +1/-1 to two-fold over/under-estimates respectively.

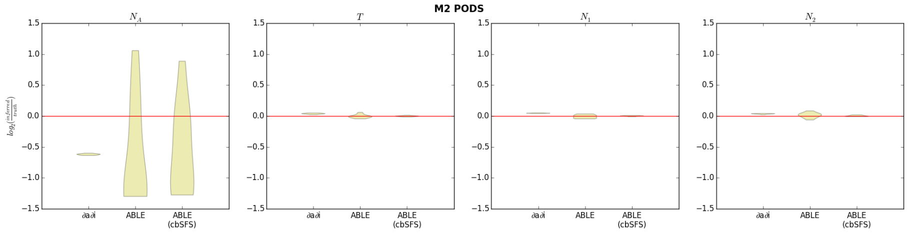

Fig. S10 Comparison of ∂a∂i and ABLE under model M6. The y-axis represents inference accuracy as given as the base-2 logarithm of the relative error (*i.e.* estimated value divided by the truth): 0 (red line) corresponds to the truth, increments of +1/-1 to two-fold over/under-estimates respectively.

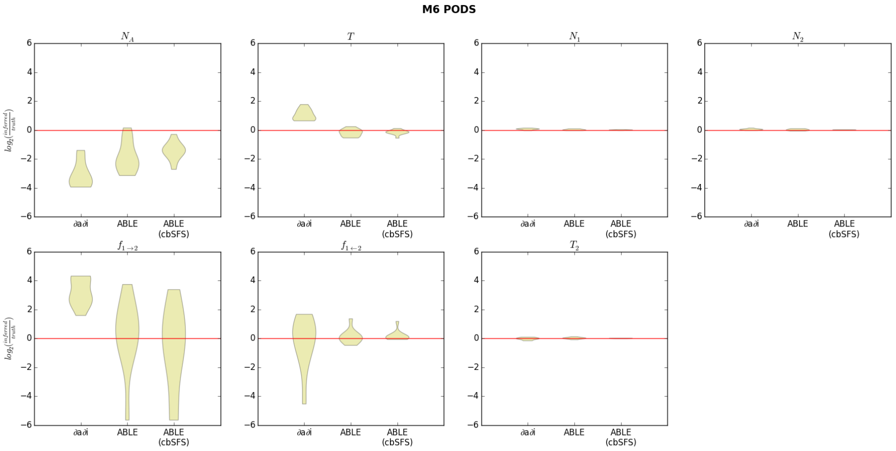

Fig. S11 Effect of long range linkage on the absolute model fit using simulations and for the most common configurations. Two datasets were simulated under M6 using long (0.5 Mb) contiguous sequences totaling 200 Mb (cut into 500bp and 2kb blocks respectively) and with the inferred MCLEs from the orangutan dataset (Table S1 and Table 1 respectively). The expected bSFS (x-axis) was generated by sampling 50 million ARG's at the inferred MCLEs and using the same values as for the simulated bSFS (y-axis). The diagonal black line indicates the perfect match between the expected and simulated. The colours represent the total number of SNPs contained in each configuration.

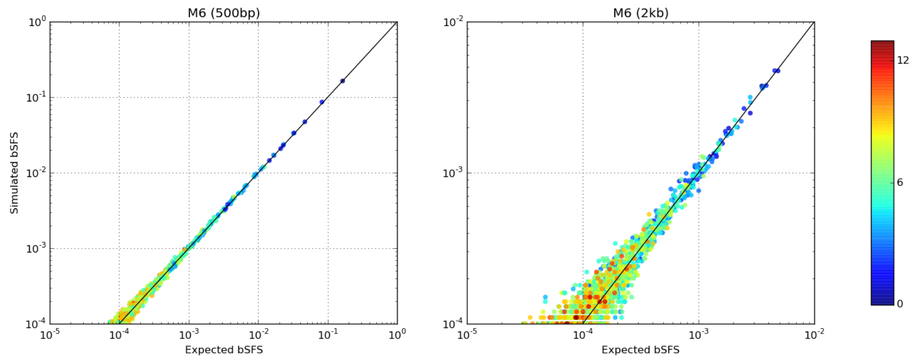

Fig. S12 Absolute model fit to the observed 500bp bSFS for the most common configurations. Each point represents a unique mutational configuration making up the bSFS. The expected bSFS (x-axis) was generated with ABLE using 50 million ARG's at the MCLE for each model (Table S1) and plotted against the observed bSFS (y-axis) from the orangutan data. The diagonal black line indicates the perfect match between the expected and observed. The colours represent the total number of SNPs contained in each configuration.

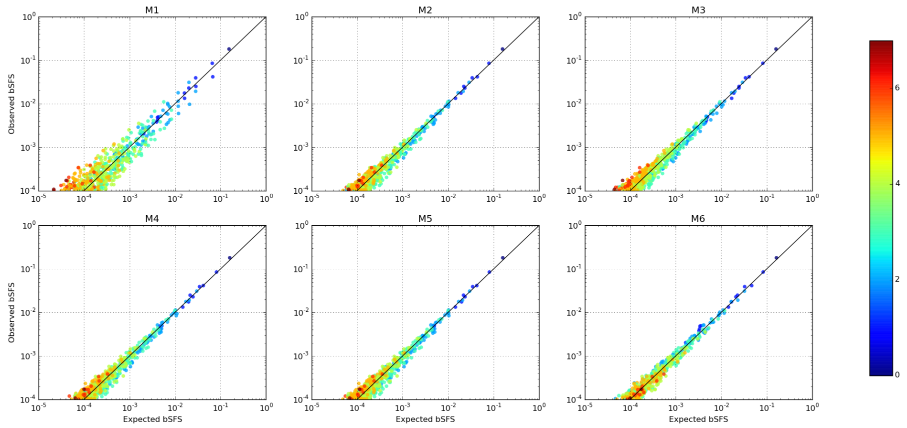

Fig. S13 The number of unique bSFS configurations for different block sizes. Data sets of a total length of 200Mb sized were simulated as independent blocks of a fixed length (x-axis) under model M6 estimated from the 2kb MCLE data (see Table 1). For each block length the number of unique bSFS configurations is plotted on the y-axis. For this history, the number of configurations defined by the bSFS is maximized for blocks between 1kb - 10kb.

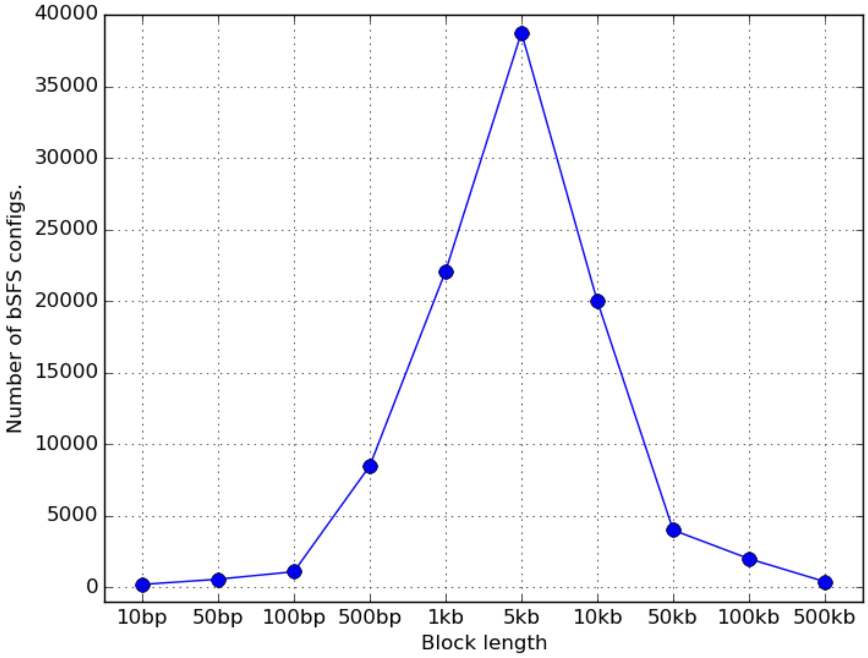

Fig. S14 Accuracy of the log likelihood (LnL) approximation for M6. The LnL was evaluated at the MCLEs obtained for the 500bp (Table S1) and 2kb datasets (Table 1) for an increasing number of sampled ARGs (x-axis). Violin plots show the entire spread around the respective averages (red dots) over 100 log likelihood evaluations. Note that the y-axis represents the *per block* CLs *i.e.* downscaled with respect to the number of blocks found in each dataset.

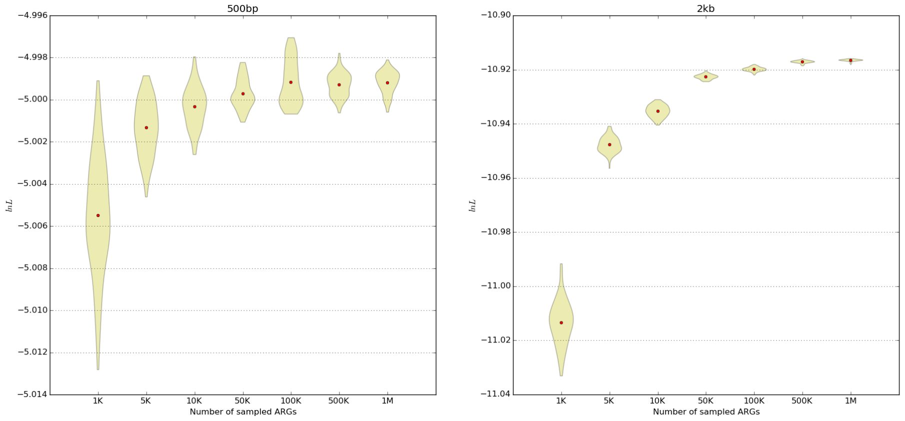

Fig. S15 Relative fit between models using 500bp blocks. Histograms of 100 evaluations of the Composite Likelihood (CL) using 1 million ARG's and at the MCLE corresponding to each model (Table S1). Note that the x-axis represents the *per block* CLs *i.e.* downscaled with respect to the number of blocks found in each dataset. Models further to the right are a relatively better fit than those to the left. Model M1 is ignored (appears much further to the left) as it has the worst fit among all models.

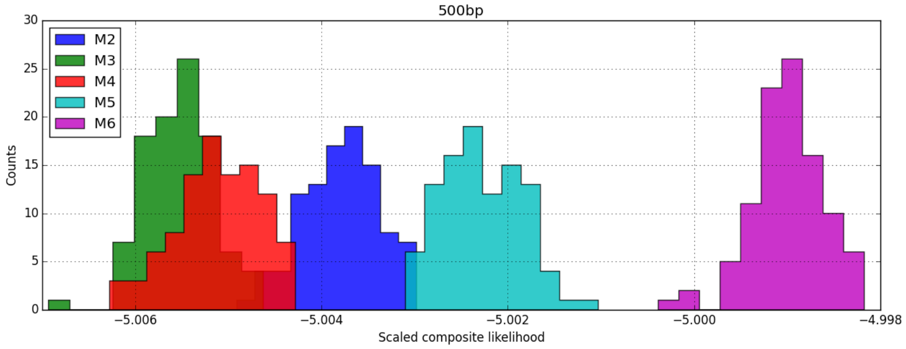

Supplement: Supplementary file 1 — Figures S1–S15. Supplementary figures. (PDF 1193 kb) [file 13059_2018_1517_MOESM1_ESM.pdf]
