# Supplementary material for: ABLE: blockwise site frequency spectra for inferring complex population histories and recombination
Source: Genome Biol. 2018 Sep 25;19:145. doi: 10.1186/s13059-018-1517-y (PMC6156964; doi:10.1186/s13059-018-1517-y)
Supplement: Supplementary file 2 — Tables S1–S5. Supplementary tables. (PDF 120 kb) [file 13059_2018_1517_MOESM2_ESM.docx]

ABLE: blockwise site frequency spectra for inferring complex population histories and recombination

Supplementary tables S1 - S5

Table S1 Point estimates for the demographic history of orangutan species obtained from 500bp blockwise data (*cf.* Fig. 4).

Model	N_A	$r \times 10^{-8}$	T	N_S	N_B	α_S	α_B	$4N_A m_{S \to B}$	$4N_A m_{S \leftarrow B}$	T_2	$f_{S \to B}$	$f_{S \leftarrow B}$	lnL
M1	18 000	3.34	413 000										-1 653 152
M2	1 640	4.68	314 000	21 900	9 090								-1 632 879
M3	1 970	4.39	347 000	23 000	4 120	0.019	-1.132						-1 632 852
M4	1 920	5.81	317 000	21 900	8 020			0.027	0.000				-1 633 028
M5	1 800	5.13	2 800 000	21 200	8 530			4.961	6.148	289 000			-1 631 767
M6	1 480	8.67	1 345 000	22 500	9 500					329 000	0.010	0.167	-1 628 548

Table S2 95% confidence intervals obtained via a parametric bootstrap. 100 datasets were simulated given the point estimates of the 500bp analysis and model M6 (Table S1). Bootstrap replicates were generated by cutting long (0.5 Mb) contiguous sequences into 500bp blocks. The confidence intervals were calculated as 2 standard deviations on either side of the maximum likelihood estimate.

Parameter	MCLE ± 2SD
N_A	1,160 - 1,810
$r \times 10^8$	5.8 - 11.5
T	822,000 - 1,869,000
N_S	19,400 - 25,700
N_B	8,100 - 10,890
T_2	285,000 - 374,000
$f_{S \to B}$	0 - 0.06
$f_{S \leftarrow B}$	0.08 - 0.26

Table S3 Point estimates under M6 (*cf.* Fig. 4) on simulations of 500bp blocks and following a sampling scheme of 1, 2 or 3 diploid genomes per population. Additionally, a single gene conversion (referred to as GC) scenario was simulated with a crossover to non-crossover rate at 1 and mean conversion tract length at 400bp. The MCLE for each sampling scheme is shown in gray, the true values shared among all the simulations are shown in white.

Model	N_A	$r \times 10^{-8}$	T	N_S	N_B	T_2	$f_{S \to B}$	$f_{S \leftarrow B}$
M6	1 250	2.50	1 000 000	16 250	8 750	500 000	0.200	0.500
M6 (1dp)	1 352	12.2	1 732 345	13 343	8 404	439 427	0.389	0.295
M6 (2dp)	1 588	2.89	1 080 289	16 360	9 055	502 330	0.283	0.426
M6 (3dp)	1 857	2.77	1 095 628	15 655	8 598	492 806	0.177	0.446
M6 (2dp, GC)	3 084	7.51	1 078 446	15 981	7 904	496 643	0.02	0.45

Table S4 True values for generating datasets under the M1, M2 and M6 models. Simulations were performed with the values specified below for studying model misspecification under progressively nested models (*cf.* Fig. 4). r stands for the recombination rate/base pair/generation and we assumed 20 years/generation. All event times (T and T_2) have been specified in years.

Model	M1	M2	M6
N_A	15,000	15,000	15,000
r	2×10^{-8}	2×10^{-8}	2×10^{-8}
T	500,000	500,000	500,000
N_1	-	15,000	10,000
N_2	-	5,000	5,000
$f_{1\to 2}$	-	-	0.05
$f_{1\leftarrow 2}$	-	-	0.3
T_2	-	-	300,000

Table S5 Point estimates for the demographic history of orangutan species obtained from revised 500bp and 2kb blockwise data (*cf.* Fig. 4). These estimates were obtained by excluding (using a cbSFS) one of the Sumatran individuals (KB9258, female) which likely belongs to a newly identified species (Nater *et. al.*, 2017). For this inference, the average genome-wide recombination rate was fixed to 2×10^{-8}/bp/generation in order to offset the loss of information from the aforementioned individual.

Model	N_A	T	N_S	N_B	T_2	$f_{S\to B}$	$f_{S\leftarrow B}$
M6 (2kb)	1 579	1 010 944	12 636	6 186	293 546	0.017	0.240
M6 (500bp)	2 961	584 897	13 353	7 213	296 137	0.602	0.612
